# A novel pure data-selection framework for day-ahead wind power forecasting

**DOI:** 10.1016/j.fmre.2021.09.011

**Published:** 2021-10-27

**Authors:** Ying Chen, Jingjing Zhao, Jiancheng Qin, Hua Li, Zili Zhang

**Affiliations:** a School of Economics and Management, Harbin Institute of Technology, Harbin 150001, China; b School of Computer Science and Technology, Harbin Institute of Technology, Harbin 150001,China; c Department of Mechanical and Industrial Engineering, Texas A&M University-Kingsville, Texas 78363, USA

**Keywords:** Day-ahead wind power forecasting, Data selection, Design and analysis of computer experiments, Heuristic optimization, Numerical weather prediction data

## Abstract

Numerical weather prediction (NWP) data possess internal inaccuracies, such as low NWP wind speed corresponding to high actual wind power generation. This study is intended to reduce the negative effects of such inaccuracies by proposing a pure data-selection framework (PDF) to choose useful data prior to modeling, thus improving the accuracy of day-ahead wind power forecasting. Briefly, we convert an entire NWP training dataset into many small subsets and then select the best subset combination via a validation set to build a forecasting model. Although a small subset can increase selection flexibility, it can also produce billions of subset combinations, resulting in computational issues. To address this problem, we incorporated metamodeling and optimization steps into PDF. We then proposed a design and analysis of the computer experiments-based metamodeling algorithm and heuristic-exhaustive search optimization algorithm, respectively.Experimental results demonstrate that (1) it is necessary to select data before constructing a forecasting model; (2) using a smaller subset will likely increase selection flexibility, leading to a more accurate forecasting model; (3) PDF can generate a better training dataset than similarity-based data selection methods (e.g., *K*-means and support vector classification); and (4) choosing data before building a forecasting model produces a more accurate forecasting model compared with using a machine learning method to construct a model directly.

## 1 Introduction

### 1.1 Overview

The sustainable and large-scale development of wind energy has received growing attention worldwide in recent years. According to the Global Wind Energy Council, the total installed global wind power capacity will reach 840 GW by 2022. The rapid development of wind energy is expected to reduce environmental pollution from traditional resources such as coal and gas; however, the uncertainties and intermittencies of natural wind bring new challenges to power-grid stability. Therefore, accurate estimation of wind power generation, especially day-ahead generation, appears critically important [1].

In the current power system, wind power producers must submit wind farms’ estimated power generation to the day-ahead market. To obtain accurate forecasting results, most relevant research [2], [3], [4] has relied on numerical weather prediction (NWP) data to develop day-ahead forecasting models via machine learning techniques. Examples include Gaussian process regression (GPR) [5], support vector regression (SVR) [6], and kernel extreme learning machine [7]. Several recent studies [8], [9], [10] have used deep neural networks to build wind power forecasting (WPF) models, considering these networks’ excellent performance in learning data features and data patterns. By contrast, others have turned to data mining methods, such as clustering and classification techniques, to distinguish among NWP data by dividing an entire dataset into several subsets based on within-data similarities. For instance, Dong et al. [11] and Gao et al. [12] exploited *K*-means to cluster NWP data and built a forecasting model for each cluster. Xiao et al. [13] sought to identify an optimal size for regression and classified a large sample set into small sets based on wind power output with different class labels. In addition, Peng et al. [14] divided NWP data dynamically into three types according to wind speed changes over a given period to predict wind power generation by type. As noted, NWP data are generated through weather research forecasting simulation models or other numerical models. Even though these models are developed well, we still can observe the phenomenon that at some time periods, low NWP wind speeds correspond to high wind power generations. In other words, these NWP data are not very accurate. However, to the best of our knowledge, these inaccurate NWP data have not drawn close scholarly attention and are often directly applied in WPF modeling processes [11,12,14,15]. As such, some necessary steps should be taken to filter out inaccurate data and further refine input data for the development of a day-ahead WPF model.

Researchers have proposed different data-selection methods to select useful data and build more accurate forecasting models in certain fields. For example, when developing short-term power system load forecasting models, Pereira et al. [16] applied a Bayesian data-selection approach to a training dataset. Bento et al. [17] used a bat algorithm for data selection. More recently, Nasseri et al. [18] developed a semi-supervised training data-selection algorithm for enhanced accuracy when forecasting seizures in canines. As demonstrated, adding data-selection steps prior to modeling can improve forecasting accuracy; however, the essence of these data-selection algorithms remains constrained to the similarities in historical data (i.e., as in the above-mentioned data mining methods). As such, existing techniques are not considered pure data-selection algorithms in this study. Each NWP data point can typically be regarded as a vector containing five elements: humidity, wind speed, wind direction, temperature, and air pressure. If the Euclidean distance of any two NWP data points is zero or quite small, then these data points are presumed to be identical or similar. In practice, identical or similar NWP data may correspond to different actual wind power outputs; using similar NWP data in one subset could thus eventually lead to an inaccurate model fit. Accordingly, relying on similarities in NWP data can generate serious WPF errors. A *pure data-selection* process is therefore defined in this study as selecting data solely based on these data's contributions to forecasting accuracy (irrespective of data similarities). This method is recommended to reduce the consequences of internal NWP data inaccuracies in forecasting models.

Specifically, we propose a novel data-selection framework as a pure data-selection process. In brief, the entire training dataset can be regarded as an array of data subset combinations. Our aim is to select useful data subsets in order to build a more accurate WPF model. However, to develop such a framework, data-selection processes may encounter a phenomenon similar tothe curse of dimensionality that occurs in the state space of dynamic programming [19]. For instance, when a training dataset consists of one-year NWP data, there are 365 subsets when using daily data as a subset. Under an exhaustive search (ES), one would need to build 2^365^ models to determine the best combination of subsets that produce the most accurate predictions; this scenario would lead to intractable computations (e.g., when considering weekly data as a subset, there would still be 2^52^ subset combinations). In other words, the ES method is not suitable for cases containing a moderate or large number of subsets. Alternatively, we may consider heuristic algorithms, such as a genetic algorithm (GA), particle swarm optimization (PSO), or Monte Carlo tree search (MCTS, [20]), to initiate potential solutions that converge to the optimal solution after a certain number of iterations. Compared to the ES method, heuristic algorithms can save substantial computational time. However, prior to direct application, two issues should be taken into account.

First, to evaluate whether a subset combination is suitable, we need to use the selected data to construct a forecasting model and assess its accuracy. In our preliminary experiment, constructing a forecasting model via a machine learning method such as SVR often took 30–60 s. Therefore, for any heuristic algorithm, it is impossible to obtain a converged solution within a reasonable time if the number of subsets is moderate or large. More importantly, due to the uncertainties and seasonal effects of wind, it is impossible to use the same WPF model to forecast wind power generation throughout an entire year. A growing number of researches, such as Feng et al. [21], have chosen to adopt a moving window–based data-updating algorithm to update the forecasting model within a certain period. We refer to this practice as the “dynamic model updating method”. Yet implementing a heuristic-based pure data-selection process with dynamic model updating is infeasible.

Second, even if the computational problem discussed above can be addressed well, nearly all heuristic algorithms are notorious for finding the local optimal solution. Some studies [22,23] have developed hybrid heuristic algorithms to improve the solution quality. For example, Juang [24] proposed a hybrid of GA and PSO for recurrent network design. Bhattacharjee and Pant [25] introduced a hybrid of PSO and GA to train a multi-layer perceptron to classify human glioma from molecular brain neoplasia data. Kim and Ahn [26] developed a hybrid fighting game with artificial intelligence using GA and MCTS. However, compared to the ES method, these hybrid algorithms can still potentially generate local optimal solutions. Therefore, based on a hybrid framework, the question of how to integrate the ES method with heuristic algorithms to further improve the optimization accuracy requires careful investigation. By bridging these knowledge gaps, we make the following contributions to the day-ahead WPF community.

### 1.2 Contribution

In this study, we use machine learning techniques to build the day-ahead WPF model with NWP data as inputs and actual wind power generation as outputs. As reported in previous report [27], wind conditions on consecutive days are moderately correlated. For day-ahead WPF, we can use this pattern to implement a data-selection process. Briefly, we consider data from the days prior to the forecasting day to generate a validation set, and then we employ the remaining historical data as training data. In a pure data-selection framework (PDF), the validation set is important because we can observe how a subset contributes to forecasting accuracy. Thus, the first contribution of this study lies in its establishment of a PDF for day-ahead WPF.

Moreover, we prefer to use a small subset to increase selection flexibility under the PDF. However, doing so can result in computational intractability, even with heuristic algorithms, as noted above. In the literature, the design and analysis of computer experiments (DACE; [28,29]) method has been applied to address the curse of dimensionality that occurs in the state space of a stochastic dynamic programming problem [30,31]. The essence of the DACE method is to use an experimental design to sample the state space and then build a metamodel with a statistical modeling method to represent the state space. With the metamodel, we can find the maximum/minimum point in the state space by using an optimization algorithm, similar to response surface methodology. Hence, the second contribution of this study involves its development of a DACE-based metamodeling algorithm to address the computational concern arising in the pure data-selection process.

The ES method is well suited to finding the optimal point in the metamodel. However, for a large-scale problem, trillions of enumerations still result in computational intractability. Using heuristic algorithms with the metamodel can conserve substantial computational time but may identify local optimal solutions. In this study, given the success of hybrid heuristic algorithms in the literature, we aim to use this approach to design an algorithm by integrating it with the ES method. Thus, the third contribution of this study is the introduction of a novel hybrid optimization algorithm—the so-called heuristic-ES optimization (HEO) algorithm—to improve optimization quality.

Our experiments can be divided into three parts. First, we sought to investigate how the HEO algorithm performed on the metamodel versus other algorithms such as GA, PSO, and MCTS. Second, we considered the use of different-sized subsets under the PDF. As mentioned, if the number of subsets is small, the ES method can be used directly without metamodeling methods. For example, if we have one-year historical data in the training dataset and use seasonal data as a subset, then we only have 2^4^ subset combinations. Therefore, such an experiment can demonstrate the necessity of proposing the DACE-based metamodeling algorithm and HEO algorithm because they are targeted to cases involving small-sized subsets. Third, we compared the forecasting results of the proposed PDF-X (*X* could be any mature machine learning method) algorithm and several forecasting algorithms presented in the literature. As mentioned before, in the WPF literature, some researchers have used data mining methods to extract important data features and improve forecasting accuracy; others have relied on machine learning methods to learn internal data patterns and enhance models’ forecasting accuracy. Such a comparison will likely reveal the core merits of this study: focusing on data selection should improve WPF forecasting accuracy more than the existing algorithms. Overall, the contributions of this study are summarized below:

(1) We proposed a novel PDF integrating metamodeling and optimization steps for day-ahead WPF.

(2) We proposed a DACE-based metamodeling algorithm to address the computational issue occurring in the pure data-selection process.

(3) We proposed an HEO algorithm to improve the metamodel's optimization quality.

(4) We performed comparison experiments to demonstrate the merits of the proposed PDF.

### 1.3 Outline of this article

The remainder of this paper is organized as follows. Section 2 provides an overview of the DACE method and the sampling method applied in this study. Section 3 details the PDF as well as the metamodeling and optimization algorithm proposed in this paper. Section 4 presents a comparison of results based on different algorithms, and Section 5 describes our conclusions and directions for future research.

## 2 Background

The computational problem discussed above is the most challenging issue and represents the core of the PDF. Therefore, we introduce the DACE method in the following subsection and then briefly describe the low-discrepancy sequence, which is the sampling algorithm we used in this study.

### 2.1 Design and analysis of computer experiments

Physical and computer experiments can both be used to determine treatment variables that affect a given response and to quantify the details of the input–output relationship [32]. Physical experiments measure stochastic responses corresponding to a set of input treatment variables, whereas computer experiments yield deterministic answers for a given set of input conditions. Computer experiments have been performed extensively in diverse fields, such as climate and weather prediction [33] and stress in prosthetic devices [34]. DACE includes the design-of-experiments concept—commonly space-filling designs such as orthogonal arrays and low-discrepancy sequences—to control input settings when running computer models. When the computer model is a deterministic simulation model, a pertinent challenge involves the “statistical” interpretation of seemingly deterministic data [29]. Sacks et al. [29] modeled deterministic response output as the realization of a stochastic process, thereby providing a statistical basis when choosing input for efficient prediction. Expanding upon work by Sacks et al. [29], Chen et al. [28] later defined two basic tasks in DACE: data collection and statistical modeling. The data collection task is controlled through experimental design with the aim of using minimal data to represent the state space well. The intention of statistical modeling is not merely to fit a pre-specified model to the data but to use modeling methods that are adaptively constructed to fit the data [31].

### 2.2 Low-discrepancy sequences

The efficient generation of random numbers to be evenly distributed within a high-dimensional space is an important part of computer experiments. For all algorithms that require sampling, evenly distributed random numbers indicate a better sample distribution. Low-discrepancy sequences, as an evenly distributed sampling method, are characterized by insignificant aggregation and fast convergence and have achieved some success in solving large-scale problems [30,31]. In particular, a low-discrepancy sequence—also known as quasi-Monte Carlo [35,36]-uses a deterministic quasi-random number sequence instead of a pseudo-random number sequence to generate a low-discrepancy distribution sequence with ultra-uniform properties. As demonstrated in [37], low-discrepancy sequence sampling is likely to overcome the curse of dimensionality in the state space of dynamic programming. In mathematics, the discrepancy of a set of points, *U*, is defined as:

(1) $$D_{R}\left(U\right)=\underset{O\in J}{\sup}\left|\frac{H\left(O;U\right)}{R}-\lambda_{s}\left(O\right)\right|$$

where J is the point set in an M-dimensional ${\left[0,1\right]}^{M}$ space, O is an arbitrarily selected area in the space, H is the number of points in the region of O, R is the total number of point sets in the space, and $\lambda_{s}\left(O\right)$ is the volume of O. As indicated in Eq. 1, the more uniform the point-set distribution is, the closer the ratio of the number of points in the selected area is to the number of points in the space and volume of the area, and the closer the calculated discrepancyis to 0. Therefore, this discrepancy can be used to evaluate the uniformity of a point set generated by a sequence in the spatial distribution.

Popular low-discrepancy sequences include the Faure sequence [35], Halton sequence [38], and Sobol sequence [39]. However, when the state-space dimensionality is high, then random numbers of adjacent two-dimensional sequences in a Faure sequence or Halton sequence demonstrate high similarity [40]. In addition, the uniformity of a Halton sequence is closely related to the number of points. By contrast, a Sobol sequence can avoid the problem of high correlation in a high-dimensional space and reach convergence faster. Therefore, a Sobol sequence was applied in this study.

## 3 Pure data-selection framework

**Fig. 1:**
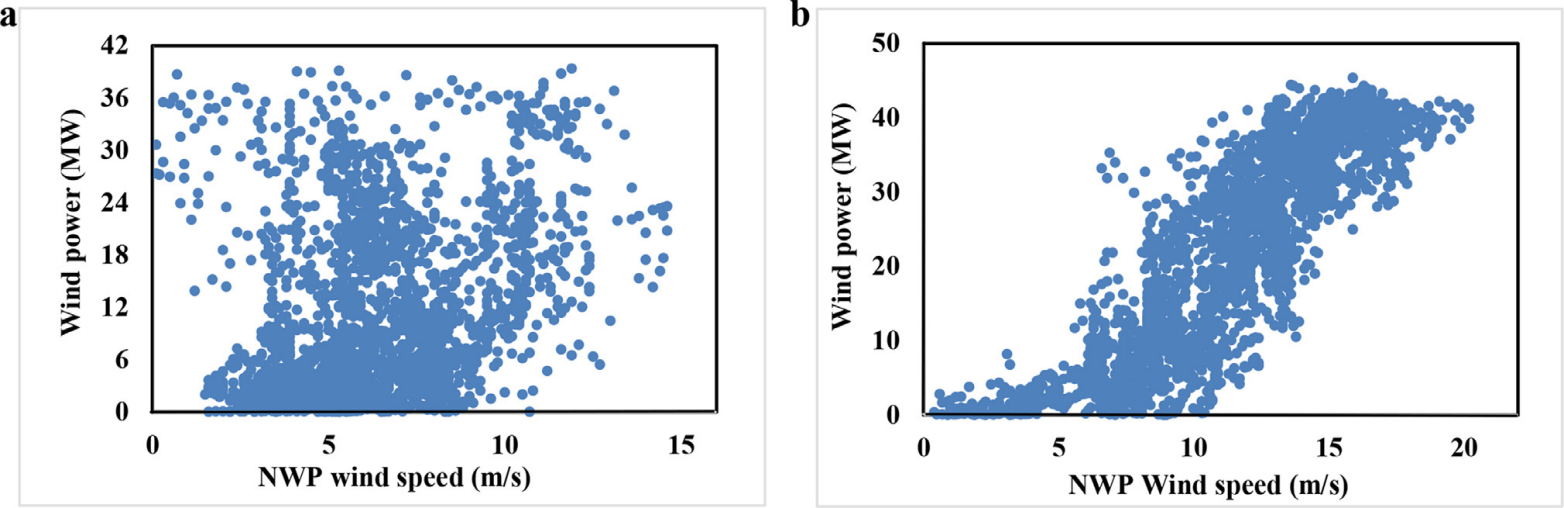
**Scatter plot of actual wind power and NWP wind speed at wind farm 1**. (a) June 2018; (b) December 2018.

As discussed above, NWP data may include inaccuracies (i.e., low NWP wind speed corresponds to high actual wind power generation). Taking data from a real wind farm in June and December 2018 as an example, the scatter plots in Fig. 1 illustrate NWP wind speed and its actual wind power.

Fig. 1a indicates that no clear pattern exists between NWP wind speed and actual wind power in this case. Most notably, low wind speeds correspond to high wind power generation in some instances. However, in Fig. 1b, wind power generation increased along with wind speed. This comparison exemplifies the occasional inaccuracy of NWP wind speed because NWP data are not actually measured data. Even though Fig. 1b appears more encouraging than Fig. 1a in terms of the relationship between wind speed and wind power, Fig. 1a, b each show that the same NWP wind speed can correspond to a large range of power generation. These patterns emphasize the importance of selecting data prior to building a forecasting model.

**Fig. 2:**
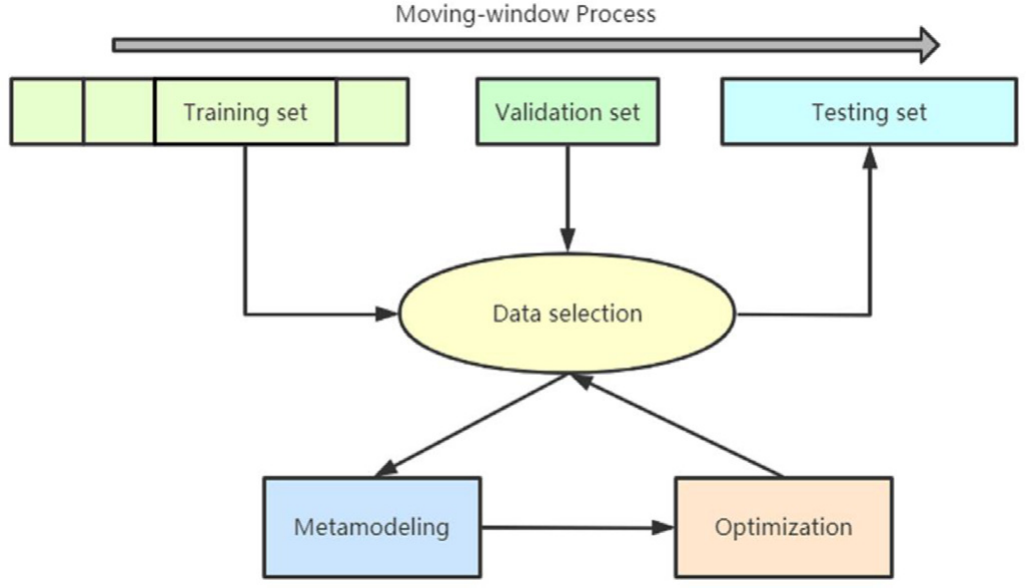
**Pure data-selection framework**.

Different from similarity-based data selection presented in [11] and [13], pure data selection defined in this study only considers the data's contributions to forecasting accuracy. A validation set is thus required to develop such a PDF. Fig. 2 details the PDF proposed in this study.

As seen in Fig. 2, the PDF is based on the moving-window process, which aims to dynamically update the training dataset and validation dataset as the testing dataset changes. In our case, the testing dataset was updated daily during day-ahead WPF. The data-selection step was implemented to select the most suitable data for the testing dataset by using the training dataset and validation dataset. Specifically, the training dataset was divided into several subsets, and then the data from each subset combination was used to build a forecasting model and examine its forecasting accuracy on the validation dataset. The subset combination with the highest forecasting accuracy was next adopted to build the forecasting model for the testing dataset. However, this process was only applicable to cases with several subsets. If the size of the subset was small (e.g., if weekly data were included in a subset), then we encountered a computational problem due to building trillions of models. As mentioned, we did not anticipate that each subset would contain a large amount of data because this scenario would compromise selection flexibility. Therefore, as seen in Fig. 2, data selection consisted of two steps: metamodeling and optimization. The metamodeling step was intended to build a metamodel and overcome the computational issue described above. The optimization step was implemented after metamodeling to identify the best subset combination from the built metamodel. As noted, this PDF is not limited to day-ahead WPF; it can be extended to other forecasting cases involving similar characteristics with which validation data can be used. In the following subsection, we describe the metamodeling and optimization steps depicted in Fig. 2.

### 3.1 DACE-based metamodeling algorithm

Inspired by Chen et al. [30] and Ariyajunya et al. [31], we incorporated the DACE method into PDF to address the computational issue. The proposed DACE-based metamodeling algorithm is detailed below and includes four tasks: subset generation, subset indication, metamodel construction, and metamodel examination.

*Subset generation***:** Assuming the total available dataset T includes historical NWP data and corresponding actual power output data from a given wind farm, then to develop a data-selection framework, we first selected a small portion from T as the validation dataset S. Assuming that each subset includes m data, a remaining dataset (T−S) containing W data, where S¬⊂W, can generateKsubsets:

(2) $$K=\frac{W}{m},K\in **Z**$$

Whether a subset is selected is represented as a binary choice. Hence, with K subsets, the available subset combinations will be $2^{K}$. In this study, we consider a large K to increase the flexibility of data selection.

*Subset indication:* We define a value N, N∈**Z**, where $N\ll 2^{K}$. Then, we apply a low-discrepancy sequence method to generate a matrix, $E_{N\times K}$; each number in this matrix is denoted as $e_{nk}$, n∈N, k∈K. Because the numbers ($e_{nk})\;$ generated via low-discrepancy sequences are between 0 and 1, we transform $e_{nk}$ numbers into binary values $b_{nk}$ and form the matrix $B_{N\times K}$:

(3) $$b_{nk}=\left\{ \begin{array}{c} 0\;\;\;\;\;\;if\;e_{nk}<0.5\\ 1\;\;\;\;\;\;\;\;otherwise\; \end{array}\right.n\in N,k\in K$$

For each sequence $\;b_{n},\;n\in N$, we assign each digit to each subset k. As such, $b_{nk}$ becomes an indicator of whether the subset is selected. That is, if $b_{nk}=1$ according to Eq. 3, then the subset is selected; the subset is not selected otherwise.

*Metamodel construction***:** We divide $B_{N\times K}$ into two sub-matrices, namely matrix $B_{P\times K}$ as the binary indication matrix for the training dataset $X^{P}$and matrix $B_{Q\times K}$ as the binary indication matrix for the testing dataset $X^{Q}$. According to each $b_{p},\;p\in P$, we use the selected subsets to generate a corresponding training dataset $X_{p}^{Train},\;p\in P$, which includes NWP data and wind power output data. Subsequently, we obtain the forecasting model $F_{p}^{Train},\;p\in P$, using $X_{p}^{Train}$, via a machine learning method. We then apply $F_{p}^{Train}$ to validation set S and achieve forecasting accuracy $A_{p},\;\;p\in P$, where $A_{p}$ is calculated using the following equation:

(4) $$A_{p}=1-\frac{RMSE_{p}}{C}$$

where C is the nominal capacity of a wind farm, and $RMSE_{p}$ indicates the root mean squared error (RMSE) of $F_{p}^{Train}$. Note that RMSE is calculated using Eq. 7 presented in Section 4. Similarly, we can obtain $A_{q}$ using the approach described above.

As mentioned earlier, it is impossible to identify the best option from all subset combinations. Hence, with the help of a machine learning method, we can build a metamodel $F_{meta}$, using $B_{P\times K}$ as input and $A_{P\;}$as output. Because N is a random number, whether N is large enough to provide sufficient data for metamodel construction is unknown; a metamodel examination is thus required.

**Fig. 3:**
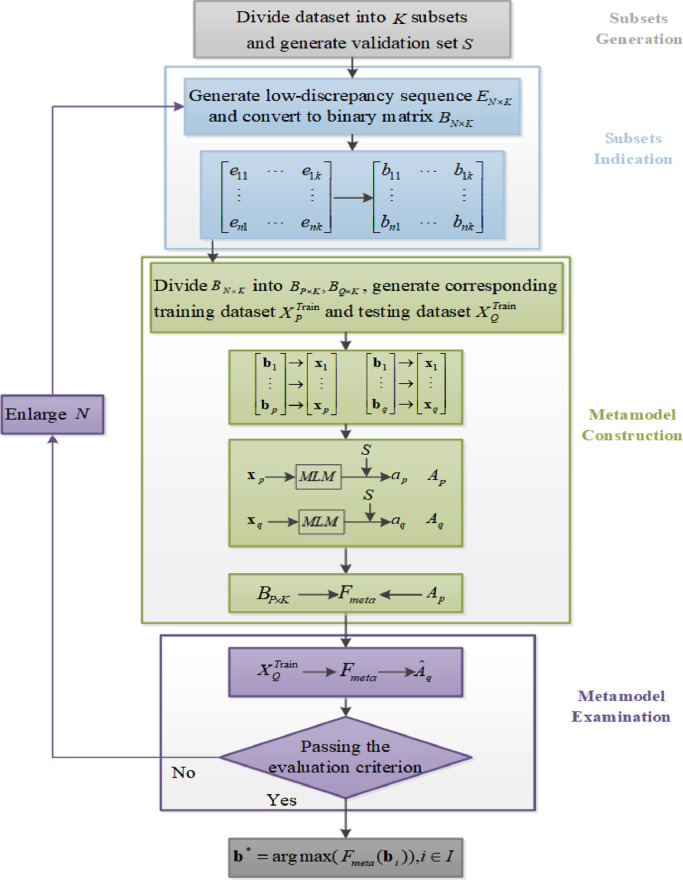
**Flowchart of metamodel construction algorithm**. (“MLM” refers to machine learning methods.)

*Metamodel examination***:** We enter $B_{Q\times K}$ into $F_{meta}$ and obtain output ${\hat{A}}_{Q}$ to examine the quality of $F_{meta}$. After comparing $A_{Q}$ and ${\hat{A}}_{Q}$ with statistical evaluation criteria, we can determine whether $F_{meta}$ is accurate. If not, then we should enlarge N and re-conduct the step in “Subset Indication”; otherwise, we can output the optimal binary string with the following equation:

(5) $$\begin{array}{c} b^{*}=\text{argmax}\left(F_{meta}\left(b_{i}\right)\right),i\in I\\ s.t.\;\;b_{i}=\left[b_{i1},\ldots b_{ik}\right]\\ b_{i1},\ldots b_{ik}=0\;or\;1\; \end{array},$$

where I denotes the total sequences, equal to $2^{K}$. Eq. 5 indicates that metamodel $F_{meta}$ is akin to a response surface; therefore, the optimization in Eq. 5 shows that finding the maximum point in the surface can reveal the optimal binary string (i.e., optimal subset combination). The detailed optimization process is presented in Section 4. To better illustrate this metamodeling process, a flowchart for the proposed DACE-based metamodeling algorithm is shown in Fig. 3. Note that once we obtain the optimal binary string, we would have the selected training data that are used to build the forecasting via a machine learning method.

The evaluation criteria in Fig. 3 are as follows: (1) the difference between consecutive tests of $R^{2}$ (i.e., the coefficient of determination) should be less than 0.005, and (2) simultaneous tests of $R^{2}$ should be greater than 0.8. Among these criteria, a difference of less than 0.005 when consecutively testing $R^{2}$ guarantees that the number of points used to build the metamodel is sufficient. A test of $R^{2}$ that is greater than 0.8 indicates that the metamodel fits the data well. We use 0.8 in this case due to the issue of model overfitting [41]. Initially, N is set to 2000 at a 2:1 proportion for training and testing, respectively, such that P = 1333 and Q = 667. If *F_meta_* does not meet the evaluation criterion, the size of N increases by 1000. Subsequently, N becomes 3000, and P and Q are updated accordingly. Due to inaccuracies in the NWP data, in an experiment, adding more sampling points will not improve the accuracy of *F_meta_* consistently. Hence, we terminate sequential sampling when N = 7,000. In addition, we consider weekly data in a subset: m is set at 672 (the wind power generation is recorded every 15 min, such that one week consists of 672 data points). Because we have two-year data for each wind farm, we make K equal to 52 (one year contains 52 weeks) to guarantee at least one year of data for selection.

### 3.2 Heuristic-ES optimization algorithm

ES is an ideal method to identify the best subset combination from the built metamodel in Eq. 5. However, considering the computational problem, direct applications remain limited. Using heuristic algorithms can save much computational time, but these alternatives may identify local optimal solutions. Hence, we proposed a hybrid algorithm by integrating heuristic algorithms with the ES method to improve the optimization quality of Eq. 5.

Briefly, we used different heuristic algorithms to generate solutions for Eq. 5. Even though heuristic algorithms can potentially return local optimal solutions, their solutions should have some similarities. In other words, a subset that possesses a unique pattern to increase forecasting accuracy should have a high probability of being selected (or not) by all heuristic algorithms simultaneously. The first task of the HEO algorithm is to identify such subsets step by step. When the subset space is reduced to one to which the ES method can be applied directly, the first task is completed.

Specifically, we define a string $b_{f}^{t}$ to represent the string containing the selection information of identified subsets at the $t^{th}$ step. Initially, $b_{f}^{0}$ is empty. The best solution string, $b_{best}^{0}$, is assumed to be an all-“1” string and the corresponding forecasting accuracy is $A_{best}^{0}$. In our case, when t=1, we used MCTS, GA, and PSO to generate a solution for Eq. 5 based on $b_{f}^{0}$, respectively. Next, we compared the four forecasting accuracies to identify the best one, $A_{best}^{1}$, which was equal to max{$A_{best}^{0}$, $A_{MCTS}^{1}$, $A_{GA}^{1}$ and $A_{PSO}^{1}$}. We could also have the corresponding best solution string, $b_{best}^{1}$, according to $A_{best}^{1}$. Then, we compared $b_{best}^{1}$, $b_{MCTS}^{1}$, and $b_{GA}^{1}$ with $b_{PSO}^{1}$ to identify subsets that were simultaneously selected or not by these four algorithms, thus outputting $b_{f}^{1}$. When t=2, following the procedure in Step 1, we implemented MCTS, GA, and PSO on $b_{f}^{1}$, which returned $A_{best}^{2}$ and $b_{best}^{2}$. Subsequently, we compared the four solutions to achieve $b_{f}^{2}$. The subset identification step did not cease until the number of unidentified subsets allowed for application of the ES method. An example of such a procedure with the HEO algorithm appears below.

**Fig. 4:**
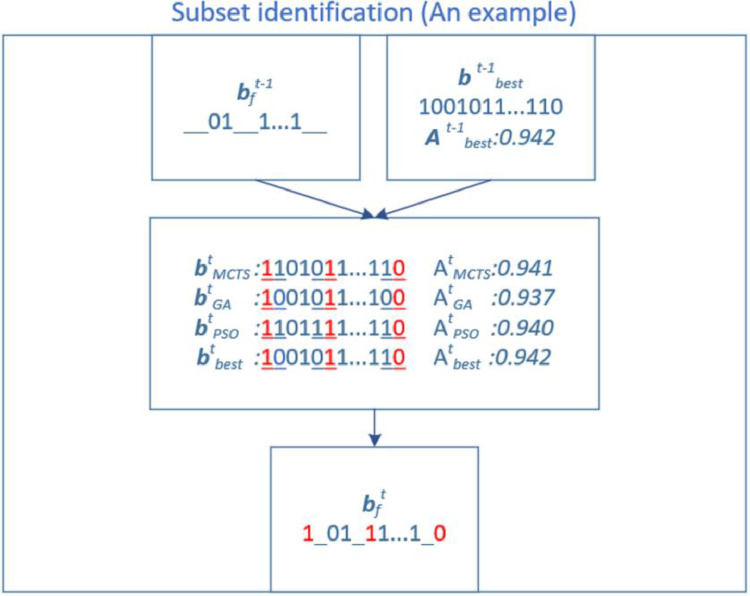
**An example of the subset identification procedure using HEO algorithm**.

In Fig. 4, the same elements at Step t in these four solution strings are marked in red. Hence, we can obtain the $b_{f}^{t}$ based on these red elements and $b_{f}^{t-1}$. As noted, in this example, $b_{best}^{t}$ is from $b_{best}^{t-1}$ because $A_{best}^{t-1}$ is better than $A_{MCTS}^{t}$, $A_{GA}^{t}$, and $A_{PSO}^{t}$. However, if $A_{best}^{t-1}$ is worse than $A_{MCTS}^{t}$, $A_{GA}^{t}$, and $A_{PSO}^{t}$, then $b_{best}^{t}$ represents the ideal option among $b_{MCTS}^{t}$, $b_{GA}^{t}$, and $b_{PSO}^{t}$. Although two solution strings are identical when identifying the subset in this case, the result is not negatively affected.Such an algorithm design can achieve a stepwise improvement in the solution quality for Eq. 5 with the following two reasons. First, these three algorithms have different searching mechanisms but are theoretically able to find the optimal solution; hence, the identified subsets should possess the properties to help the three solutions each tend toward the optimal solution. Second, we used the best string from the previous step as another solution string to compare with strings generated by the three algorithms; we were unsure whether we would be likely to further improve the solution quality due to the local optimal issue when referring only to previously identified subsets. In other words, the subsets identified in earlier steps could belong to local optimal solutions, causing the newly identified subsets to possibly lower the solution quality. Therefore, the core of this comparison procedure was to guarantee that the best solution at each step was at least not worse than the prior one.

Once the number of unidentified subsets was reduced to an appropriate figure, we adopted the ES method to enumerate all solutions by integrating the identified ones. For day-ahead WPF, we implemented the ES method when the number of unidentified subsets was between 15 and 20. Note that implementing 2^20^ = 1048576 potential solutions on the built metamodel only took roughly 3 min. The pseudo code of this algorithm 1 is presented below.

## 4 Experiments

In this section, we evaluate the PDF by applying it to three existing wind farms. We first describe our data in Section 4.1 and present the results of our three experiments in Section 4.2. Lastly, we discuss the practicality of the proposed method for the current power system in terms of computational time in Section 4.3.

### 4.1 Data description

Data from three existing wind farms were applied in this study. Basic information about these wind farms appears in Table 1. Because our predictions involved the day-ahead market, we exclusively used NWP data, which has a temporal resolution of 15 min and five types of information (i.e., humidity, temperature, wind speed, wind direction, and air pressure). Following Dong et al. [11], we used sine and cosine to preprocess the wind direction and thus included six variables in our dataset.

**Table 1:** **Basic information on wind farms used in this study**.

Wind farm (WF) no.	WF1	WF2	WF3
Number of wind turbines	33	24	24
Installed capacity (MW)	49.5	48	48
Manufacturer type	Envision EN70-1500	Vestas V80-2000	Gamesa G90
Hub height of tower/s (m)	70	80	90
Cut-in / Cut-out wind speed (m/s)	4.0 / 25.0	3.5 / 25.0	3.0 / 21.0
Rated wind speed (m/s)	11.6	14.5	11.0
Swept area of a wind turbine (m^2^)	3915	5027	6362

Due to equipment failure and maintenance, output generation may sometimes be absent or lost; hence, when more than 10% of data were missing for a specific day, all data points from that day were deleted from the dataset. Otherwise, we used the average interpolation method to fill in missing data. We considered the size of the validation set and the modeling methods used to build the metamodel. Our experiments were conducted on a computer equipped with Intel® Xeon Gold 6138 CPU at 2.00 GHz, 256 GB RAM, and 40 cores. To measure forecasting results, in line with prior literature, we used the mean absolute error (MAE) and RMSE as defined below:

(6) $$MAE=\frac{1}{M}\sum_{t=1}^{M}\left|y_{t}-{\hat{y}}_{t}\right|$$

(7) $$RMSE=\sqrt{\frac{1}{M}\sum_{t=1}^{M}{\left(y_{t}-{\hat{y}}_{t}\right)}^{2}}$$

where M is the number of forecasting periods, $y_{t}$ stands for the actual wind power at time t, and ${\hat{y}}_{t}$represents the forecasting wind power. Because the forecasting accuracy was measured every day, we set M to 96 considering the temporal resolution of 15 min in NWP data.

The focus of this study concerned the merits of the proposed PDF when using a small subset; therefore, we did not closely consider the forecasting model construction. In our preliminary experiments, no major differences tend to be observed in forecasting results when using different machine learning methods to build forecasting models with the same data. We therefore adopted SVR with radial basis function (RBF) kernel to construct forecasting models and used the 10-fold cross-validation method within SVR for model calibration and validation. Moreover, we used 3-day data before the forecasting day as the validation set; using one-day data could generate prediction bias, and using more-day data might reduce the moderate correlation between the validation dataset and the forecasting dataset.

**Fig. 5:**
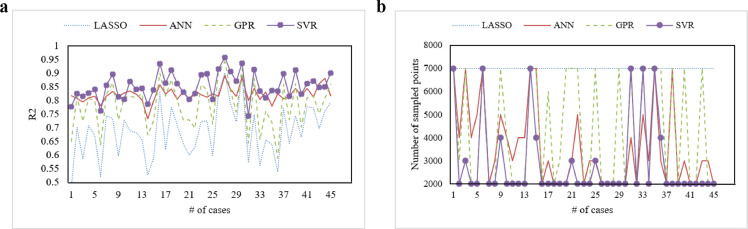
**Results comparison of four machine learning methods to build the metamodel**. (a) $R^{2}$ results for each metamodel and (b) number of sampled points used to build the metamodel.

As introduced in Section 3, the input for building our metamodel was depicted in a binary matrix, which is quite different from traditional modeling input. Although current machine learning techniques are well established, few studies have considered this type of input. Eq. 5 shows that the metamodel can reveal the optimal subset combination; metamodel quality is thus paramount. Hence, we chose four popular machine learning techniques—SVR with RBF kernel, LASSO regression (LASSO), artificial neural networks (ANN), and GPR—to build the metamodel. Day-ahead forecasting involves daily implementation. Referring to the number of cases adopted to examine the appropriateness of decision-making policies in [31] and [42], we randomly selected 15 daily data points in the year 2019 from each wind farm to produce 45 cases. Fig. 5 displays the number of sampled points in the training set and the coefficient of determination ($R^{2}$) for each metamodel in the 45 cases.

Fig. 5a shows that, the $R^{2}$ results for SVR were higher than those for the other machine learning methods in most cases: the average $R^{2}$ results for LASSO, GPR, ANN, and SVR were 0.6872, 0.7835, 0.8219, and 0.8523, respectively. Moreover, as seen in Fig. 5b, when using LASSO to build the metamodel, the 45 cases covered 7,000 points (we limited the number of maximum sampled points to 7000 considering the computational time). GPR also required 7000 points in some cases, whereas ANN and SVR included 7000 points in several cases. On average, the DACE-based metamodeling algorithm required 7000, 4288, 3555, and 2866 points to build the metamodel when using LASSO, GPR, ANN, and SVR, respectively. Based on these findings, we could apply the minimal amount of data to devise a more accurate metamodel by using SVR. We therefore adopted SVR for metamodel construction, given the binary input.

### 4.2 Experiment

In the following subsections, we describe three experiments that reflect the merits of the proposed PDF using small subsets. First, we explored the performance of the proposed HEO algorithm by benchmarking with GA, PSO, and MCTS. In the second, we included weekly, monthly, and seasonal data as respective subsets to exemplify the need to use a small subset. Lastly, we took the other algorithms as benchmarks to demonstrate the advantages of the proposed PDF in Fig. 2. As noted, in the first two experiments, we also applied the 45 cases collected above.

#### 4.2.1 Experiment 1

**Fig. 6:**
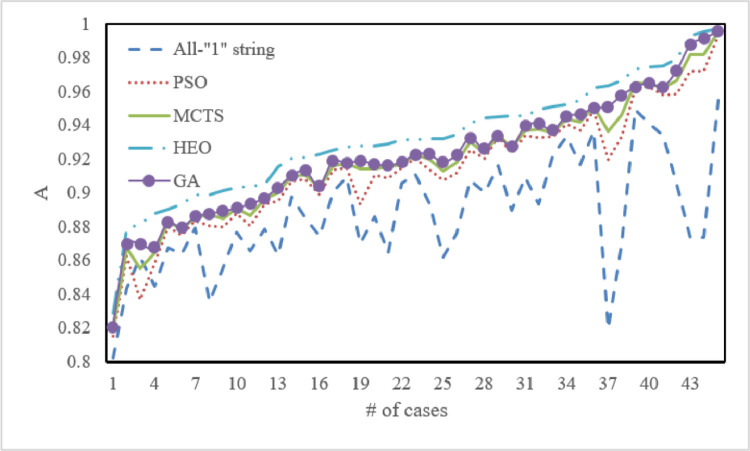
**Result comparison using different algorithms for Eq.**5.

In this subsection, we explain how the proposed HEO algorithm performed in identifying the optimal point in the metamodel. First, we took the algorithm that selected all subsets (i.e., the binary string was an all-“1” string) as a benchmark to demonstrate the need to develop the PDF. In addition, we used GA, PSO, and MCTS as other benchmarks to indicate the optimization quality of the HEO algorithm. Regarding the parameters of GA, PSO, and MCTS, we simply applied the default values in each toolbox because the results did not change drastically after tuning. The results are shown in Fig. 6, where Ais the forecasting accuracy introduced in Eq. 4.

The all-“1” string resulted in the lowest forecasting accuracy compared with the other four algorithms, which clearly indicates the need and advantages of developing a data-selection algorithm before modeling. As to the performance of the other four algorithms, PSO, MCTS, GA, and HEO exhibited a highly similar trend in Fig. 6; however, HEO seemed to outperform the other three alternatives in all cases. In addition, as shown in Fig. 6, the gaps between using the all-“1” string and the heuristic algorithms (e.g., PSO, GA, and MCTS) were much larger than the variation when using these three heuristic algorithms and the HEO algorithm. This outcome indicates that data selection played a relatively greater role in improving the forecasting model's accuracy than in identifying the optimal point on the metamodel using the HEO algorithm.

Furthermore, we carried out a statistical analysis using a paired *t*-test to demonstrate the outstanding performance of HEO. Results are listed in Table 2. All p-values were less than 0.05, indicating that HEO generated statistically different results from the other four algorithms. Additionally, all confidence intervals (CIs) were greater than 0; that is, HEO statistically managed to improve the optimization quality more than a single heuristic algorithm. Moreover, the proposed HEO algorithm was not only intended to find the high-quality solution in the metamodel; it could also be applied to other problems with discrete variables. Some examples include the wind farm layout optimization problem, in which a wind farm can be divided into several squares [43, 44].

**Table 2:** **Statistical analysis of forecasting accuracy comparison between HEO and four other algorithms (**α**= 0.05)**.

	HEO vs. All-“1” string	HEO vs. PSO	HEO vs. MCTS	HEO vs. GA	
$p_{A}$^a^	<0.000	< 0.000	< 0.000	< 0.000	
	$CI_{A}$	(0.0368, 0.0538))	(0.0164, 0.0213)	(0.0120, 0.0150)	(0.0094,0.0117)

a p indicates the pvalue of paired *t*-test, and CIindicates the confidence interval

#### 4.2.2 Experiment 2

**Fig. 7:**
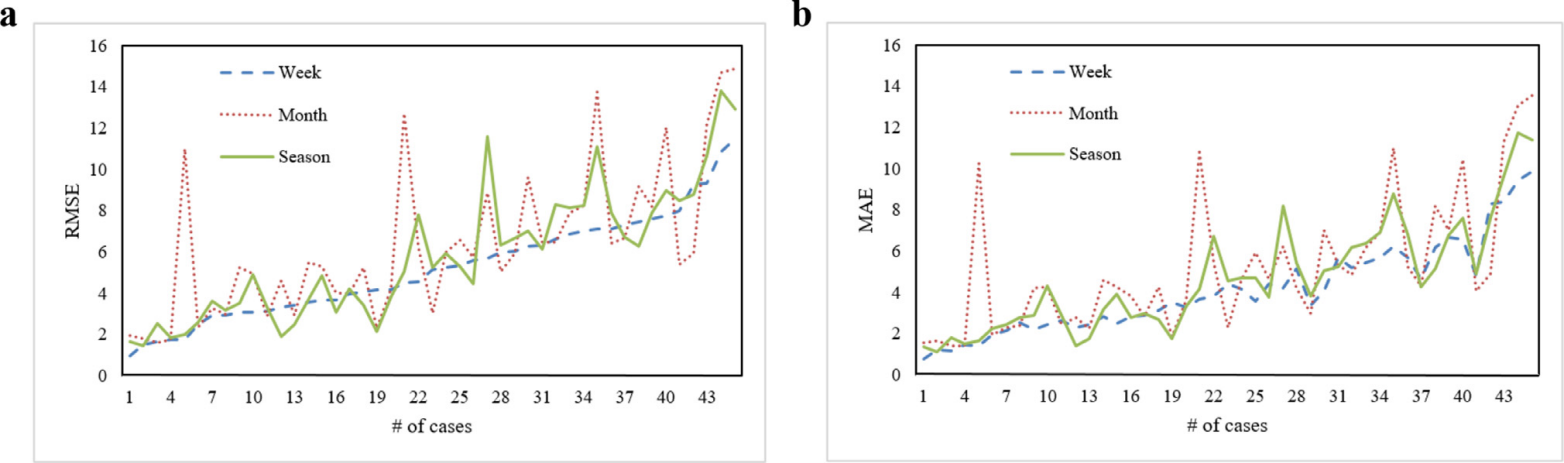
**Forecasting accuracy comparison using weekly, monthly, and seasonal data in a subset**. (a) Results using RMSE; (b) results using MAE.

In Section 3, we outlined a data-selection framework in which data are chosen based on their forecasting contributions to the validation set. As noted, a smaller subset size is associated with greater flexibility in data selection; however, whether such flexibility can increase forecasting accuracy requires further investigation. In this subsection, we presented a comparison experiment consisting of weekly, monthly, and seasonal data as subsets to demonstrate the need for the proposed algorithm. As noted, when using monthly and seasonal data in a subset, the metamodeling and optimization steps in the PDF were unnecessary because there were only 2^12^ = 4096 subset combinations and 2^4^ = 16 subset combinations, respectively. We could thus select the best subset combination using the ES method within a reasonable time for these two cases. The testing set also contained the 45 cases used in the above experiments. SVR with RBF kernel was used to approximate the forecasting model for these three scenarios; forecasting accuracy results are illustrated in Fig. 7. Notably, one selection candidate of these three cases comprised all subsets, indicating that all data in the initial training dataset would be used. However, all results in Fig. 7 did not come from this candidate; this result corresponds to the outcome when using the all-“1” string in Experiment 2. In other words, data selection was required to build an accurate forecasting model even when using a large subset.

**Fig. 8:**
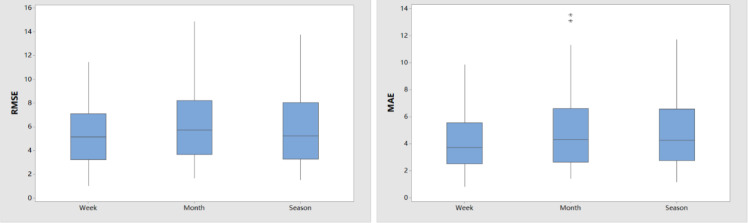
**Boxplot of RMSE and MAE results using weekly, monthly, and seasonal data in a subset**.

**Fig. 9:**
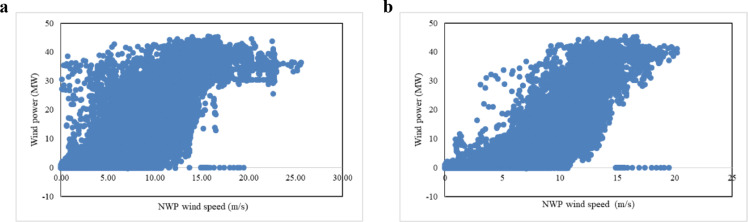
**Scatter plot of NWP wind speed and actual wind power**. (a) Without using any data-selection method; (b) with using the proposed PDF method.

The forecasting accuracy when incorporating these three types of data into a subset was similar in some cases. Fig. 7 shows that no clear pattern could indicate which subset size was better. However, using monthly or seasonal data in a subset could sometimes lead to poor forecasting accuracy (e.g., Cases 5, 21, and 35). Hence, we conducted a statistical analysis of RMSE and MAE values for these 45 cases using a paired *t*-test to demonstrate the general performance of the proposed PDF with small subsets; see Table 3. As indicated, upon comparing the use of weekly data in a subset to the use of monthly or seasonal data in a subset, all p-values for RMSE and MAE were less than 0.05, and all CIs were less than zero. These outcomes demonstrate that using a smaller subset can enhance the forecasting model's accuracy. We also generated a boxplot to display the RMSE and MAE values in Fig. 8, which shows that the RMSE and MAE results when using weekly data in a subset led to a smaller variance than the other two cases. In other words, using weekly data in a subset was likely to generate more stable forecasting accuracy. To further validate the effects of the proposed selection method, in comparison to Fig. 1, we randomly selected one case and illustrated the relationship between NWP wind speed and corresponding wind power generation in Fig. 9. Despite some inaccurate data in Fig. 9b, the proposed PDF was likely to eliminate many inaccurate data points. Additionally, because we used the weekly data in a subset, it was impossible to eliminate all inaccurate data with the proposed PDF. All in all, Table 3 and Figs. 8, 9 showcase the benefits of using a smaller subset for the PDF.

**Table 3:** **Statistical analysis of RMSE and MAE results (**α**= 0.05)**.

	Week vs. Month	Week vs. Season	Month vs. Season	
$p_{RMSE}$^a^	0.003	0.008	0.095	
	$CI_{RMSE}$	(-1.898, -0.417)	(-1.002, -0.159)	(-0.105, 1.259)
$p_{MAE}$^a^	0.002	0.002	0.066	
	$CI_{MAE}$	(-1.764, -0.439)	(-0.857, -0.214)	(-0.038, -1.170)

a p indicates the p-value of paired *t*-test, and CI indicates the confidence interval

#### 4.2.3 Experiment 3

To further examine the performance of the proposed PDF with small subsets, we chose four algorithms from the literature as benchmarks. The first was from [11], who used K-means and ANN to forecast day-ahead wind power (we name this method as ANN*_k_*); the second was from [13], who introduced a method to categorize data via support vector classification and then used SVR to build a forecasting model for each subset (we name this method as SVR*_c_*); the third algorithm was from [45], who found that a single-input-multiple-output (SIMO)–based deep learning structure was likely to lead to a better forecasting model; the fourth was from [46], who introduced an improved long-short-term memory (ILSTM) network to forecast wind power. The first two benchmarking algorithms that involve data mining prior to modeling can be considered representative of similarity-based data selection methods. The other two algorithms, which use popular deep learning techniques to acquire internal data features and build high-quality forecasting models, are reflective of focusing directly on the construction of a forecasting model. This comparison aims to demonstrate the merits of pure data selection for day-ahead WPF compared with existing methods. We named the algorithm integrating the PDF to select data and the X machine learning method to build the forecasting model the “PDF-X” algorithm. As SVR was used to build the forecasting model, we employed PDF-S to represent our algorithm as follows.

In the algorithm comparison experiment, past studies [47,48] often used data from four seasons for investigation. Therefore, in this subsection, we randomly select 10-day data as a test set in each season. The forecasting accuracy over four seasons is listed in Table 4 with the best accuracy per case in bold.

**Table 4:** **Forecasting accuracy comparison between PDF-S and existing algorithms by season**.

	**Spring**	PDF-S	ANN*_k_*	SVR*_c_*	SIMO	ILSTM		**Summer**	PDF-S	ANN*_k_*	SVR*_c_*	SIMO	ILSTM
WF1	RMSE	8.3939	9.8152	**8.3404**	8.7834	8.4321	WF1	RMSE	**5.3415**	5.7635	6.6873	6.6333	6.4323
	MAE	**6.5745**	7.6654	6.8614	7.0823	6.7666		MAE	**4.2214**	4.428	5.4019	5.4039	5.6321
WF2	RMSE	**6.4365**	8.4459	8.1897	8.0452	7.8992	WF2	RMSE	**5.779**	8.6355	7.0624	7.1298	8.1568
	MAE	**5.1678**	6.8012	6.8735	6.6728	6.3423		MAE	**4.5775**	6.7804	5.3671	5.3399	6.0391
WF3	RMSE	8.3005	**7.7459**	10.9417	7.9322	8.0523	WF3	RMSE	**5.3394**	7.4176	5.4887	6.0728	6.3923
	MAE	6.5178	**5.9291**	8.5296	6.2113	6.5432		MAE	**4.1418**	5.4802	4.2009	4.8332	4.9988
	**Autumn**	PDF-S	ANN*_k_*	SVR*_c_*	SIMO	ILSTM		**Winter**	PDF-S	ANN*_k_*	SVR*_c_*	SIMO	ILSTM
WF1	RMSE	**5.3634**	6.4703	6.5349	7.3537	7.0328	WF1	RMSE	**6.439**	8.9269	7.9067	6.7923	7.0135
	MAE	**4.5746**	5.216	5.5427	5.9393	5.7845		MAE	**5.5524**	7.0435	6.8718	5.6982	5.8091
WF2	RMSE	**3.8349**	5.2443	7.284	4.7378	5.0254	WF2	RMSE	6.9223	**6.8793**	6.9513	7.0812	7.2112
	MAE	**3.0723**	3.9107	6.3908	3.5014	3.5924		MAE	5.7521	**5.3971**	5.7234	5.631	5.7398
WF3	RMSE	**8.6784**	9.8182	11.082	9.5208	9.4046	WF3	RMSE	**5.7559**	7.5334	8.176	6.3988	6.2934
	MAE	**6.4156**	7.1665	9.6498	6.9364	6.8902		MAE	**4.639**	6.0156	6.9682	5.1982	5.0921

# ANN*_k_*, SVR*_c_*, SIMO, and ILSTMrepresent the methods used in [11, 13, 45], and [46], respectively; the best forecasting accuracy is highlighted in bold for each wind farm

As shown in Table 4, although ANN*_k_*and SVR*_c_*each performed better in several cases,the proposed PDF-S algorithm outperformed the others in terms of MAE and RMSE in most cases. To more clearly convey the quality of the PDF-S algorithm, we referred to the accuracy difference percentage:

(8) $$D=\frac{Z-S}{Z}\times 100\%$$

where *Z* represents the overall forecasting accuracy (i.e., RMSE or MAE) of one of the four existing algorithms, and *S* represents the overall forecasting accuracy (i.e., RMSE or MAE) of the PDF-S algorithm; results appear in Table 5 As observed, for $D_{RMSE}$ (i.e., the accuracy difference percentage of RMSE), the PDF-S algorithm performed better than the other four from 6.18% to 22.09% for the three wind farms. Regarding $D_{MAE}$, the PDF-S algorithm outperformed the others from 6.86 to 26.01%. These findings demonstrate the advantages of the proposed PDF-S algorithm over possible alternatives. Most importantly, Table 5 indicates that focusing on data selection before modeling is more effective than focusing on model construction [45, 46] to improve the accuracy of day-ahead WPF.

**Table 5:** **Overall accuracy difference percentage of forecasting accuracy between PDF-S algorithm and four other algorithms**.

		PDF-S vs. ANN*_k_* (%)	PDF-S vs. SVR*_c_* (%)	PDF-S vs. SIMO (%)	PDF-S vs. ILSTM (%)
WF1	$D_{RMSE}$	17.56	13.34	13.61	11.67
	$D_{MAE}$	14.08	15.22	13.27	12.79
WF2	$D_{RMSE}$	21.34	22.09	14.90	18.80
	$D_{MAE}$	18.87	23.75	12.18	14.48
WF3	$D_{RMSE}$	13.66	21.34	6.18	6.86
	$D_{MAE}$	11.70	26.01	6.32	7.69

**Algorithm 1:** **Heuristic-ES optimization algorithm**.

Initialize: *t*← 0, *b*${}_{f}^{t}$←*None, b*${}_{best}^{t}$← [1111...11], $A_{best}^{t}$
Set *t* = 1
*b*${}_{MCTS}^{t}$, *b*${}_{GA}^{t}$, *b*${}_{PSO}^{t}$←*MCTS* (*b*${}_{f}^{t-1}$), *GA* (*b*${}_{f}^{t-1}$), *PSO* (*b*${}_{f}^{t-1}$)
$A_{MCTS}^{t}$, $A_{GA}^{t}$, $A_{PSO}^{t}$←$F_{meta}$(*b*${}_{MCTS}^{t}$), $F_{meta}$(*b*${}_{GA}^{t}$), $F_{meta}$(*b*${}_{PSO}^{t}$)
$A_{best}^{t}$ = *max* {$A_{best}^{t-1}$, $A_{MCTS}^{t}$, $A_{GA}^{t}$, $A_{PSO}^{t}$}
*b*${}_{best}^{t}$ = $\begin{array}{c} argmax\\ b \end{array}$($F_{meta}$(*b*)), *b* ∊ {*b*${}_{best}^{t-1}$, *b*${}_{MCTS}^{t}$, *b*${}_{GA}^{t}$, *b*${}_{PSO}^{t}$}
*b*${}_{f}^{t}$←*subset identification* (*b*${}_{f}^{t-1}$, *b*${}_{best}^{t-1}$, *b*${}_{MCTS}^{t}$, *b*${}_{GA}^{t}$, *b*${}_{PSO}^{t}$).
If the number of unidentified subsets satisfied the solvable range of the ES method:
return ES (*b*${}_{f}^{t}$)
Else *t* = *t* + 1, and go to Step 2.

### 4.3 Further discussion

According to local power system regulations, wind power producers should submit their day-ahead power estimates to the power system prior to 8:00 AM. In other words, after obtaining next-day NWP data, wind power producers have 8 h at most to complete power forecasting. The PDF-S algorithm (we can use other machine learning methods to build the forecasting model) in Experiment 3 only took roughly 2.5 h on average to obtain the forecasting model. Hence, we may consider using smaller subsets versus weekly data. In our further exploration, forecasting accuracy did not differ significantly when using weekly versus 6-day data in a subset; by contrast, the forecasting accuracy improved when using 5-day data or even less in a subset. However, the computational time had already exceeded 8 h on average when using 5-day data. Therefore, for day-ahead WPF, we recommend using weekly data with the PDF-*S* algorithm.

## 5 Conclusion

NWP data for day-ahead WPF contain internal inaccuracies as shown in Fig. 1. Even though data mining and machine learning methods have achieved some success in day-ahead WPF, these inaccurate data have not been addressed and continue to influence modeling processes. In this study, we proposed a PDF to select useful data before modeling. To compensate for the computational issue and obtain a high-quality data-selection result, we developed a DACE-based metamodeling algorithm and HEO algorithm, which we incorporated into the PDF, as described in Section 3.

We conducted four experiments to illustrate our findings. In the first experiment, we discovered that SVR with RBF kernel was most appropriate for building a metamodel with binary input. In the second experiment, we exploited four benchmarking algorithms to validate the outstanding performance of the HEO algorithm.In the third experiment, we used monthly and seasonal data in separate subsets as benchmarks to show that using weekly data in a subset can generate better overall forecasting accuracy. We then chose four existing algorithms as benchmarks to demonstrate the superiority of the PDF-*S* algorithm in terms of forecasting accuracy. Specifically, we came to the following conclusions: (1) it is necessary to select data before constructing a forecasting model; (2) using a smaller subset will likely increase selection flexibility, leading to a more accurate forecasting model; (3) a PDF can generate a better training dataset than similarity-based data-selection methods (e.g., *K*-means and support vector classification); and (4) focusing on data selection before building a forecasting model results in a more accurate forecasting model compared with focusing on model construction directly. All in all, our comparison results underline the need for and advantages of the proposed PDF.

The average computational time for each data-selection process using weekly data as a subset was roughly 2.5 h. Although this computational time is still long, it remains reasonable because wind power producers need to submit day-ahead WPF results before 8:00 AM; they have 8 h to build a forecasting model for the next day. We tried to use the subset containing 5-day data; however, the computational time would be more than 8 h on average using the current computational facility. Therefore, in the future, scholars should focus on further reducing computational time to enable use of a smaller subset with 5-day data or even less. Doing so should increase data-selection flexibility. Besides these, an adaptive stopping criterion for the sequential sampling algorithm should be investigated. In addition, other experimental methods (e.g., an orthogonal array and Latin hypercube sampling) can be adopted for comparison with low-discrepancy sequences. Lastly, researchers could adopt the proposed PDF to establish forecasting models for wind speed, load, or solar power.

## Declaration of competing interest

The authors declare that they have no conflicts of interest in this work.
